# Atezolizumab, bevacizumab, pemetrexed and platinum for *EGFR*‐mutant NSCLC patients after EGFR TKI failure: A phase II study with immune cell profile analysis

**DOI:** 10.1002/ctm2.70149

**Published:** 2024-12-23

**Authors:** Shang‐Gin Wu, Chao‐Chi Ho, James Chih‐Hsin Yang, Shu‐Han Yu, Yen‐Feng Lin, Shu‐Chin Lin, Bin‐Chi Liao, Ching‐Yao Yang, Yen‐Ting Lin, Chong‐Jen Yu, Ya‐Ting Chuang, Wei‐Yu Liao, Kah Yi Yap, Weng Si Kou, Jin‐Yuan Shih

**Affiliations:** ^1^ Department of Internal Medicine National Taiwan University Cancer Center Taipei Taiwan; ^2^ Department of Internal Medicine National Taiwan University Hospital Taipei Taiwan; ^3^ Department of Oncology National Taiwan University Cancer Center Taipei Taiwan; ^4^ Department of Oncology National Taiwan University Hospital Taipei Taiwan; ^5^ Graduate Institute of Oncology Cancer Research Center National Taiwan University Taipei Taiwan; ^6^ Institute of Biotechnology National Taiwan University Taipei Taiwan; ^7^ Center for Neuropsychiatric Research National Health Research Institutes Miaoli Taiwan; ^8^ Department of Public Health & Medical Humanities School of Medicine National Yang Ming Chiao Tung University Taipei Taiwan; ^9^ Institute of Behavioral Medicine College of Medicine National Cheng Kung University Tainan Taiwan; ^10^ Department of Medical Research National Taiwan University Hospital Taipei Taiwan; ^11^ Department of Internal Medicine National Taiwan University Hospital Hsinchu Branch Hsinchu Taiwan

**Keywords:** anti‐angiogenesis, atezolizumab, EGFR TKI resistance, immune cell, tumour microenvironment

## Abstract

**Background:**

Acquired resistance to epidermal growth factor receptor tyrosine kinase inhibitors (EGFR TKIs) remains a significant hurdle for patients with *EGFR*‐mutated non‐small cell lung cancer (NSCLC), particularly those lacking the *EGFR^T790M^*. IMpower 150 study demonstrated promising efficacy for a combination of immune‐chemotherapy and bevacizumab in patients with *EGFR*‐mutated NSCLC.

**Methods:**

This open‐label, single‐arm, phase II trial evaluated the efficacy and immune cell profile of the modified regimen combining atezolizumab, bevacizumab (7.5 mg/kg) and chemotherapy in patients with *EGFR*‐mutated NSCLC following TKI failure. The primary endpoint was objective response rate (ORR). The re‐biopsy tissue specimens and serial peripheral blood samples were collected to analyse the immune cell profile and tumour microenvironments.

**Rresults:**

22 *EGFR*‐mutant NSCLC patients participated in this study. The ORR was 42.9%, with a disease control rate (DCR) of 100%. Median progression‐free survival (PFS) was 6.3 months. Patients with programmed death‐ligand 1 (PD‐L1) expression ≥1% exhibited significantly higher ORR (75 vs. 23.1%; *p* = .032) and longer PFS (14.0 vs. 6.1 months; *p* = .022) compared with those with PD‐L1 expression < 1%. Grade ≥ 3 adverse events occurred in 40.9% of patients. Higher peritumour nature killer (NK) cell infiltration and lower peripheral helper T cell counts before treatment were associated with favourable ORR and longer PFS, respectively. After disease progression, the proportion of S100A9^+^ myelod‐derived suppressor cells (MDSCs) increased, while regulatory T cells decreased.

**Conclusion:**

This modified combination regimen may be a promising therapeutic option for *EGFR*‐mutant NSCLC patients with TKI resistance, especially those with PD‐L1‐positive tumours. Furthermore, immune cell profiling may aid in identifying patients who may benefit from this approach.

**Key points:**

- The combination regimen yielded promising efficacy in NSCLC patients after EGFR‐TKI resistance, particularly those with PD‐L1‐positive tumours.
- Higher peritumour NK cell and lower peripheral helper T cell were associated with favourable ORR and longer PFS, respectively.
- After disease progression, the proportion of S100A9^+^ MDSC increased, but Treg cells decreased.

## Graphical abstract

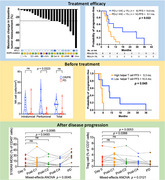

## 1 INTRODUCTION

Accounting for 80–85% of all lung cancers, non‐small cell lung cancer (NSCLC) remains a leading cause of cancer mortality. While targeted therapies have revolutionised precision medicine for NSCLC, resistance to first‐ and second‐generation epidermal growth factor receptor tyrosine kinase inhibitors (EGFR TKIs) typically emerges within 9–14 months, often due to the *EGFR^T790M^* mutation.^1^, ^2^, ^3^, ^4^ The third‐generation EGFR TKIs such as osimertinib effectively target *EGFR^T790M^* mutation along with common mutations,^5^ yet disease progression ultimately occurs.^6^ Salvage platinum‐based therapy achieves only a 10–20% response rate,^7^, ^8^ highlighting the urgent need for innovative strategies, especially for patients with acquired resistance to EGFR TKIs.

Immune checkpoint inhibitors (ICIs), particularly anti‐programmed death‐1 (PD‐1)/programmed death‐ligand 1 (PD‐L1) antibodies, have demonstrated efficacy in various solid tumours,^9^, ^10^, ^11^, ^12^ including NSCLC. However, as single agents, they have shown limited efficacy in *EGFR*‐mutated NSCLC, possibly due to low tumour mutational burden (TMB), lower PD‐L1 expression and an immunosuppressive tumour microenvironment (TME) in these patients.^13^, ^14^ Nevertheless, re‐biopsy samples after acquired resistance to EGFR TKIs exhibited increasing PD‐L1 and TMB,^13^, ^15^ suggesting the potential suitability of ICIs for this population.

Following disease progression on first‐line EGFR‐TKI therapy, Yoo et al. reported a 5.4‐month progression‐free survival (PFS) and a 34.8% objective response rate (ORR) with the combination of pemetrexed and cisplatin. However, this combination therapy did not demonstrate superior efficacy to pemetrexed monotherapy in terms of response rate, PFS or overall survival (OS). Therefore, there remains a need to identify optimal treatment regimens, including potential combination strategies with ICIs. The phase III trials, KEYNOTE‐789 and CheckMate‐722 trials, showed trends towards improved survival with ICIs and chemotherapy in patients with TKI‐resistant *EGFR*‐mutated NSCLC, but they lacked statistical significance.^16^, ^17^ In contrast, IMpower 150 demonstrated favourable responses with atezolizumab, bevacizumab and chemotherapy for this patient population, irrespective of PD‐L1 expression.^12^, ^18^ Furthermore, the phase III APPLE (WJOG11218L) clinical trial revealed that the incorporation of anti‐angiogenic agents into immune‐chemotherapy regimens resulted in an extended PFS for patients with *EGFR*‐mutated NSCLC after EGFR‐TKI failure (HR, 0.70; 95% CI, 0.46–1.06).^19^ The bevacizumab's ability to modulate the TME towards an immunocompetent state may contribute to the enhanced efficacy of ICIs.^20^ Therefore, several phase II and III clinical trials (e.g., NEJ043, APPLE (WJOG11218L), IMpower 151, ORIENT‐31 and ATTLAS (KCSG‐LU19‐04), etc.) have explored the therapeutic potential of combining anti‐angiogenic agents with immune‐chemotherapy in patients with *EGFR‐*mutated NSCLC who have progressed on EGFR TKIs.^18^, ^19^, ^21^, ^22^, ^23^, ^24^ These studies have yielded diverse results in terms of ORRs and PFS. The factors contributing to these variations in clinical outcomes remain elusive and require further investigation.

Prompted by the high prevalence of *EGFR* mutations in East Asian NSCLC patients and the challenges in managing EGFR‐TKI‐resistant cases,^25^ we investigated a modified IMpower 150 regimen substituting pemetrexed for paclitaxel and administering bevacizumab at 7.5 mg/kg every 3 weeks to optimise tolerability and minimise toxicity.^26^ Re‐biopsy tissue samples and peripheral blood were collected to explore dynamic alterations in immune cell profiles and the TME, potentially revealing biomarkers.

## 2 RESULTS

### 2.1 Patient characteristics

Between April 2020 and March 2022, 30 patients with lung cancer were screened at National Taiwan University Hospital (NTUH). Of these, 22 met eligibility criteria and were enrolled. Data analysis was conducted with a cutoff date of 10 November 2023, resulting in a median follow‐up duration of 18.7 months.

Detailed patient characteristics were presented in Table 1. The median age was 63.5 years (range 46–73). A total of 14 patients (63.6%) were female, and 16 (72.7%) were non‐smokers. Prior to enrolment, nine patients received first‐generation EGFR TKIs (gefitinib/erlotinib), five received afatinib and eight received osimertinib (including one gefitinib/osimertinib, two erlotinib/osimertinib and two afatinib/osimertinib). Of the 22 patients enrolled in the study, 11 (50%) had a history of prior radiotherapy. Irradiated sites included lung lesions (*n* = 2), brain lesions (*n* = 2), bone lesions (*n* = 1), combined lung and bone lesions (*n* = 2), combined brain and bone lesions (*n* = 3) and combined lung and brain lesions (*n* = 1). At study entry, eight patients (36.4%) presented with brain metastases. Two patients had one metastatic sites, four patients had two metastatic sites and 16 patients (72.7%) had three or more metastatic sites (Table 1).

**TABLE 1:** Clinical characteristics of the enrolled patients.

	Patients *n* (%)	
Total	22	(100.0%)
Age, median, years	63.5	
(range)	(46–73)	
Sex		
Female	14	(63.6%)
Male	8	(36.4%)
Smoking status		
Non‐smokers	16	(72.7%)
Smokers	6	(27.3%)
Histology		
Adenocarcinoma	20	(90.9%)
Poorly‐differentiated carcinoma	2	(9.1%)
EGFR mutation		
Del‐19	9	(40.9%)
L858R	11	(50.0%)
Other	2	(9.1%)
Prior EGFR TKI		
Gefitinib/erlotinib	9	(40.9%)
Afatinib	5	(22.7%)
Osimertinib	8	(36.4%)
PD‐L1 IHC		
<1%	14	(63.6%)
≧1%	8	(36.4%)
Brain metastasis		
Yes	8	(36.4%)
No	14	(63.6%)
Metastatic sites (organs)		
1	2	(9.1%)
2	4	(18.2%)
≥3	16	(72.7%)
Prior radiotherapy		
Yes	11	(50.0%)
No	11	(50.0%)

All 22 patients underwent pre‐enrolment re‐biopsy of tumour tissue. The re‐biopsy methods employed included eight bronchoscopy, six video‐assisted thoracoscopic surgeries (VATS), three ultrasound‐guided, three computed tomography (CT)‐guided and two pleuroscopy biopsies. Among the re‐biopsy tissue samples, *EGFR* mutations included nine exon‐19 deletions, 11 L858R, one G719X and one L861Q (Table 1). Pre‐enrolment PD‐L1 expression was ≥1% in 8 (35.0%) re‐biopsy tumours.

### 2.2 Clinical efficacy and survival analysis

All patients received at least one dose of combination treatment. One patient was excluded from treatment response analysis due to idiopathic thrombocytopenia purpura after the first cycle. One patient remained on study treatment as of the data cutoff date of 10 November 2023.

**FIGURE 1:**
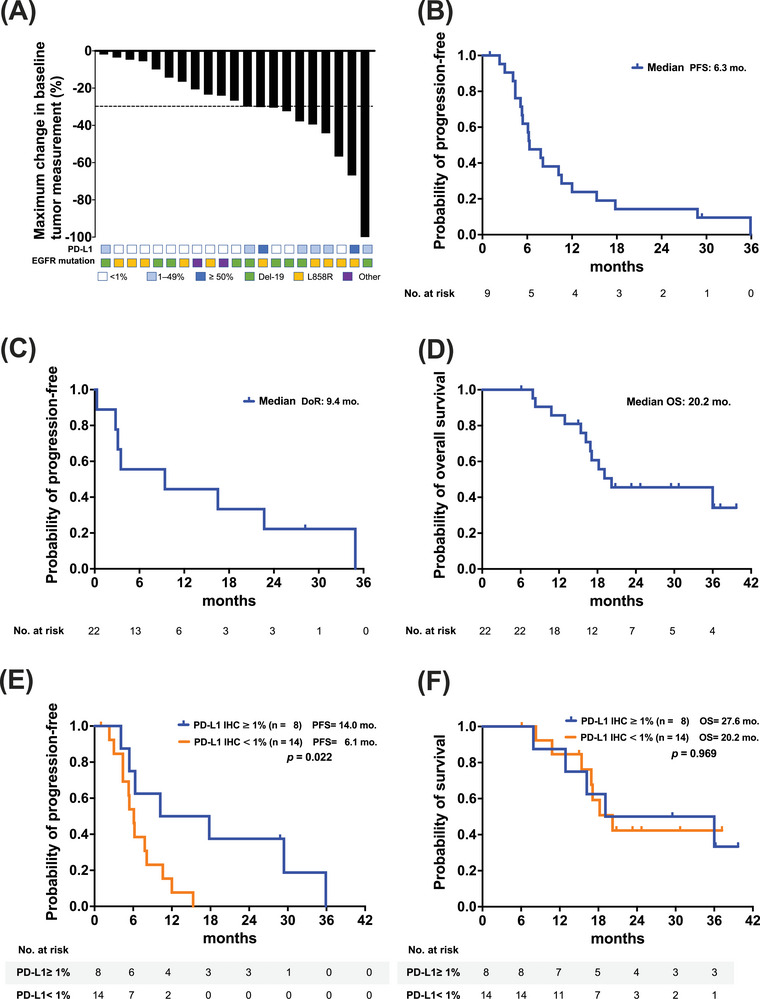
(A) Waterfall plot of the response. Best treatment response from baseline in tumour size (*n* = 21). Tumour size was defined as the sum of the longest diameters of target lesions, and the changes of tumour size were measured according to RECIST version 1.1 by investigator review at baseline and at least one follow‐up radiological assessment. Kaplan–Meier survival curve of (B) duration of response, (C) progression‐free survival and (D) overall survival in patients with *EGFR*‐mutated lung cancer who received atezolizumab, bevacizumab, pemetrexed and cisplatin or carboplatin. (E) Differences in progression‐free survival between patients with (≧1%) and without PD‐L1 expression (<1%) was statistically significant (14.0 vs. 6.1 months; *p* = .022, by the log‐rank test). (F) The difference in OS did not reach a significant difference (27.6 vs. 20.2 months; *p* = .969, by the log‐rank test).

Among the 21 evaluable patients, one achieved complete response (CR) and eight achieved partial response (PR). The overall ORR was 42.9% (95% confidence interval (CI): 21.2–64.6%), which did meet its primary endpoint of ORR. However, the disease control rate (DCR) was 100% (Table 2) The best percentage change from baseline was based on the sum of the longest diameters of target lesions on CT scans at baseline and subsequent imaging timepoints (Figure 1A). No cases of primary resistance were observed.

**TABLE 2:** Clinical characteristics of the patients enrolled for treatment efficacy analysis.^a^

		Best response				
	All patients^a^	CR+PR		SD		*p* ^b^
Total	21	9	(42.9%)	12	(57.1%)	
Age, median, years	63.5	62.0		65.5		.917^c^
(range)	(46–73)	(54–72)		(46–73)		
Sex						1.000
Female	13	6	(46.2%)	7	(53.8%)	
Male	8	3	(37.5%)	5	(62.5%)	
Smoking status						.659
Non‐smokers	15	7	(46.7%)	8	(53.3%)	
Smokers	6	2	(33.3%)	4	(66.7%)	
*EGFR* mutation						.345
Del‐19	9	5	(55.6%)	4	(44.4%)	
L858R	10	4	(40.0%)	6	(60.0%)	
Other	2	0	(0.0%)	2	(100.0%)	
Prior EGFR TKI						.268
Gefitinib/erlotinib	9	5	(44.4%)	4	(55.6%)	
Afatinib	5	2	(20.0%)	3	(80.0%)	
Osimertinib	7	2	(28.6%)	5	(71.4%)	
PD‐L1 IHC						.032
<1%	13	3	(23.1%)	10	(76.9%)	
≧1%	8	6	(75.0%)	2	(25.0%)	
Brain metastasis						.673
Yes	8	4	(50.0%)	4	(50.0%)	
No	13	5	(38.5%)	8	(61.5%)	
Metastatic sites (organs)						.611
<3	5	3	(60.0%)	2	(40.0%)	
≥3	16	6	(37.5%)	10	(62.5%)	
Prior radiotherapy						.387
Yes	11	6	(54.5%)	5	(45.5%)	
No	10	3	(30.0%)	7	(70.0%)	

Abbreviations: CR, complete response; PR, partial response; SD, stable disease.

^a^ One patient was excluded the efficacy analysis due to idiopathic thrombocytopenia purpura.

^b^ By Fisher's exact test.

^c^ By Mann–Whitney U test.

Patients with PD‐L1 expression (PD‐L1 IHC ≥ 1%) demonstrated higher response rates (75.0% [six of eight]) compared with those without PD‐L1 expression (PD‐L1 IHC < 1%) (23.1% [three of 10]; *p* = .032) (Table 2 and Figure 1A). No significant differences were observed in gender, smoking history, *EGFR* mutation types or prior EGFR TKI exposure between patients with CR/PR and stable disease (Table 2). Furthermore, there were no significant differences in response rate between patients with or without brain metastases (50.0 vs. 38.5%; *p* = .673), regardless of the number (<3 vs. ≥3: 60.0 vs. 37.5%; *p* = .611) of metastatic sites or prior radiotherapy history (yes vs. no: 54.5 vs. 30.0%; *p* = .387).

Median PFS was 6.3 months (95% CI: 3.8–8.8 months) (Figure 1B), median duration of response (DoR) was 9.4 months (95% CI: 0.0–26.6 months) (Figure 1C), and median OS was 20.2 months (95% CI: 2.2–38.8 months) (Figure 1D). There were six patients suffered from CNS progression, and the intracranial PFS was not reached (NR) (95% CI: NR–NR).

### 2.3 Subgroup analysis of PFS

Subgroup analysis was conducted to investigate factors influencing PFS. Patients with PD‐L1 expression ≥1% exhibited longer median PFS compared with those with PD‐L1 expression < 1% (14.0 vs. 6.1 months; *p* = .022) (Figure 1E and Table 3). However, OS did not differ significantly between these groups (27.6 vs. 20.2 months; *p* = .969) (Figure 1F). No significant differences in PFS were observed based on sex (*p* = .569), smoking status (*p* = .751), *EGFR* mutation type (*p* = .534) or prior EGFR TKI exposure (*p* = .228) (Table 3). In addition, there were no significant differences in PFS between patients with or without brain metastases (4.4 vs. 10.2 months; *p* = .101), regardless of the number (<3 vs. ≥3: 17.8 vs. 5.4 months; *p* = .082) of metastatic sites.

**TABLE 3:** Multivariate analysis of predictive factors for PFS in patients with non‐small cell lung cancers who received atezolizumab in combination with bevacizumab, carboplatin or cisplatin and pemetrexed after failure of EGFR tyrosine kinase inhibitors.

Factor	Number of patients	PFS (months)	Univariate analysis	Multivariate analysis	
			*p*	HR (95% CI)	*p*
Sex					
Female	14	6.3		1	
Male	8	6.2	.569	0.36 (0.88–1.43)	.355
Smoking history					
Non‐smokers	16	6.3		1	
Smokers	6	5.3	.751	0.83 (0.16–4.36)	.825
*EGFR* mutation					
Del‐19	9	6.1		1	
L858R	11	6.2		1.59 (0.39–6.50)	.522
Other	2	7.8	.534	0.12 (0.01–1.12)	.063
Prior EGFR TKIs					
Gefitinib/erlotinib	9	8.1		1	
Afatinib	5	7.8		0.25 (0.04–1.47)	.126
Osimertinib	7	6.1	.228	4.12 (0.41–41.75)	.231
PD‐L1 TPS					
<1%	14	6.1		1	
≥1%	8	14.0	.022	0.12 (0.03–0.51)	.004

Abbreviations: PFS, progression‐free survival; HR, hazards ratio; CI, confidence interval; ECOG PS, Eastern Cooperative Oncology Group performance status; PD‐L1, programmed death‐ligand 1; TPS, tumour proportion score; *EGFR*, epidermal growth factor receptor gene; EGFR‐TKI, epidermal growth factor receptor tyrosine kinase inhibitor.

Multivariate analysis using a Cox regression model was conducted to identify potential predictive factors of PFS (Table 3). This analysis confirmed that patients with PD‐L1 expression ≥1% had significantly longer median PFS compared with those with PD‐L1 expression < 1% (HR: 0.12, 95% CI: 0.03–0.51; *p* = .004).

### 2.4 Adverse events analysis

All 22 patients (100%) experienced at least one adverse event (Table S5). The majority of adverse events were grade 1 or 2, with the most frequent being abnormal liver function (31.8%) and constipation (18.2%), neutropenia (18.2%) and rash acneiform (18.2%). Grade 3 drug‐related adverse events occurred in nine patients (40.9%), predominantly abnormal liver function (9.1%). Notably, no grade 4 or 5 (fatal) adverse events were observed. There were two thromboembolic events detected, including one pulmonary embolism and one deep venous thrombosis.

### 2.5 Post‐progression re‐biopsy genetic sequence analysis and treatment

By 10 November 2023, 21 patients had experienced disease progression or death. Of these 21 patients, 13 had re‐biopsy tissue samples available after progression on the combination therapy. A re‐biopsy of only one of these 13 patients revealed a transformation to small cell carcinoma. Additionally, nine of the 21 patients underwent post‐progression comprehensive genomic profiling using next‐generation sequencing (NGS). These nine samples included six tumour tissue samples, two plasma samples and one malignant pleural effusion. NGS analysis identified a diverse range of genetic alterations, as detailed in Table S6 and Figure S1.

Eighteen patients (85.7%) received subsequent systemic therapy after disease progression. The median number of post‐progression treatment regimens was 2 (range: 0–9). Thirteen patients received various chemotherapy regimens, while 17 (81%) received rechallenged EGFR TKIs (10 erlotinib and seven osimertinib). Additionally, three patients received anti‐angiogenesis treatments, all in combination with EGFR TKIs. Rechallenge with EGFR TKIs occurred post‐chemotherapy, not immediately following immunotherapy. Grade 1/2 skin rash was observed in three (17.6%) patients, and grade 1/2 abnormal liver function, including elevated GOT/GPT in three patients and jaundice in one, was noted in four (23.5%) patients. No cases of interstitial pneumonitis were reported following EGFR TKI rechallenge.

### 2.6 Tumour infiltrating immune cell analysis

**FIGURE 2:**
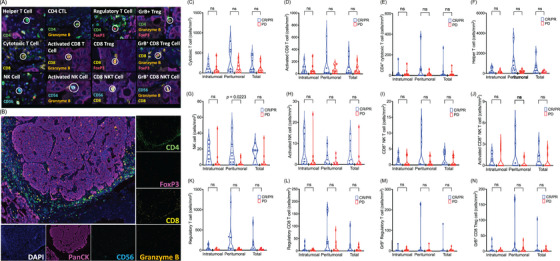
(A) Multiplex immunohistochemistry defines the precise location of immune subsets in re‐biopsy lung cancer samples (*n* = 21). FFPE sections of non‐small cell lung cancer were stained by ‘Lymphocytic Panel’ for CD4; CD56; CD8; granzyme B; FoxP3; and PanCK. The immune cell density was defined as the number of the immune cells in the area (mm^2^). Different immune cells were calculated in three areas (intratumoural, peritumoural and total). The immune cells included: (B) cytotoxic T cell, (C) activated CD8^+^ T cell, (D) CD4^+^ cytotoxic T cell, (E) helper T cell, (F) NK cell, (G) activated NK cell, (H) CD8^+^ NK T cell, (I) activated CD8^+^ NK T cell, (J) regulatory T cell, (K) regulatory CD8^+^ T cell, (L) GrB^+^ regulatory CD8^+^ T cell and (M) GrB^+^ CD8^+^ regulatory T cell. Mann–Whitney *U* test was adopted across the two groups (CR/PR vs. SD).

There were 21 re‐biopsied tumours before trial enrolment to analyse the TME using Opal multiplex immunofluorescence staining (Figure 2A,B). While no significant differences in intratumoural, peritumoural or total populations of various immune cells were observed between CR/PR and stable disease (SD) groups (Figure 2B–N), a higher median peritumoural natural killer (NK) cell density correlated with better treatment response (CR/PR: 7.65 vs. SD: 0.28/mm^2^; *p* = .0223) (Figure 2B,G).

### 2.7 Immune related biomarker analysis

**FIGURE 3:**
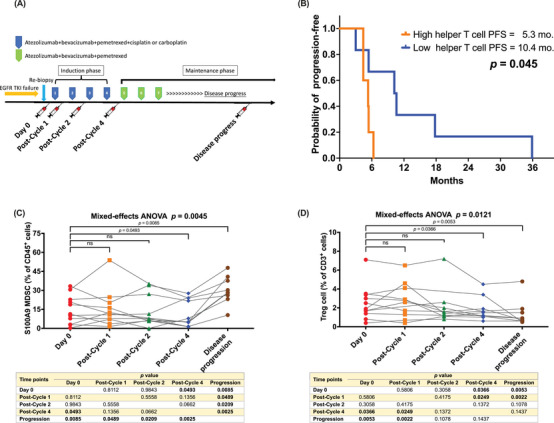
Effect of the atezolizumab, bevacizumab, pemetrexed and platinum chemotherapy on peripheral immune cells. (A) the schema of the timepoints for blood sampling in the immune biomarker exploration study. (B) Patients with a lower percentage of helper T cells before treatment was associated with longer PFS than those with a higher percentage of CD8^+^ effector cells (10.2 vs. 5.3 months; *p* = .045 by log‐rank test). (C) S100A9^+^ MDSC cells, increased after disease progression (*p* = .0045 by mixed‐effect ANOVA). The table compared the proportion of S100A9^+^ MDSC cells at two different time points using a Student's *t*‐test. (D) Treg cells significant declined in proportion after disease progression (*p* = .0121 by mixed‐effect ANOVA). The table compared the proportion of Treg cells at two different time points using a Student's *t*‐test.

Of the 22 patients, 11 joined the immune biomarker exploration study. All of them signed informed consent forms for peripheral blood or tumour tissue sample collection. The patients’ peripheral blood were collected at Day 0, post‐Cycle 1, post‐Cycle 2, post‐Cycle 4 and after disease progress (Figure 3A).

First, we explored the correlation between PFS and immune cell proportions before treatment (Day 0) based on the median percentage of peripheral immune cells. A lower percentage of helper T cells at Day 0 was correlated with longer PFS (5.3 vs. 10.4 months; *p* = .045) (Figure 3B). Additionally, we investigated the correlation between peripheral blood helper T cell counts and tumour tissue infiltration. An inverse correlation was observed between these two parameters (*R* = −0.414) (Figure S4). This finding suggests that increased infiltration of CD4^+^ T cells within the TME is associated with decreased levels of circulating helper T cells.

Then, we also explored the proportions of peripheral immune cells before treatment (Day 0) to predict treatment responses. According to the treatment responses, we divided the patients into two groups: CR/PR (one CR and five PR) and SD (*n* = 5). There were no significant differences in the proportions of the various immune cells at Day 0 between CR/PR and SD groups (Figure S2).

We also analysed the proportions of various peripheral immune cells over time points using mixed‐effects ANOVA (Figure S3). Notably, S100A9^+^ myelod‐derived suppressor cells (MDSCs), exhibited a significant increase after disease progression (*p* = .0045) (Figure 3C). Additionally, another immunosuppressive cell population, regulatory T (Treg) cell, significantly declined after disease progression (*p* = .0121) (Figure 3D).

## 3 DISCUSSION

This study assessed the effectiveness of combining atezolizumab, bevacizumab, pemetrexed and carboplatin/cisplatin for *EGFR*‐mutated NSCLC patients resistant to EGFR TKIs. While the primary endpoint of response rate was not met, the combination therapy demonstrated encouraging disease control. Positive PD‐L1 expression (≥1%) correlated with higher ORR and longer PFS. Patients achieving complete or partial response showed greater peritumoural infiltration of NK cells. A lower percentage of helper T cells before treatment was correlated with longer PFS. Dynamic changes in immune cell profiles were observed. After disease progression, the proportion of S100A9^+^ MDSCs increased, while Treg cells decreased.

Studies on combined ICIs, anti‐angiogenesis drugs and chemotherapy for NSCLC patients revealed varying response rates, ranged from 43.9 to 70.6%,^12^, ^21^, ^24^, ^27^ exceeding those observed in the current study. This discrepancy may be attributed to differences in chemotherapy backbones and bevacizumab dosing regimens employed across studies.^12^, ^18^, ^21^, ^24^, ^27^ In contrast to IMpower 150 and ATTLAS studies,^12^, ^18^ the current study utilised pemetrexed instead of paclitaxel. Furthermore, a lower dose of bevacizumab was administered in the current study compared with NEJ043 trial.^21^ While higher bevacizumab doses may potentially enhance efficacy, they are often associated with increased toxicity and reduced cost effectiveness.^26^, ^28^ Further studies are necessary to investigate the optimal chemotherapy regimen for this combination therapy approach.

The study followed the treatment regimen of Lam et al.,^24^ administering bevacizumab at 7.5 mg/kg every 3 weeks. While the observed response rate (RR) of 42.8% was lower than that reported by Lam et al.,^24^ it remained consistent with the phase III ORIENT‐31 trial.^27^ This discrepancy may stem from the limited sample size and varying PD‐L1 expression among enrolled patients' tumours. Multivariate analysis identified PD‐L1 expression as a significant predictor of both RR and PFS. Tumours with PD‐L1 expression (≥1%) exhibited a more favourable response to the immune‐chemotherapy combination. Notably, 63.6% of patients in this study had tumours lacking PD‐L1 expression (<1%), surpassing the proportion observed in Lam et al.’s study.^24^ The heterogeneity in patient populations and chemotherapy regimens likely contributed to the observed response differences.

Despite the significant PFS benefit, there was no difference in OS in patients with PD‐L1‐positive IHC. The relatively small sample size of this study limits its power to assess OS. A larger phase III trial is necessary to definitively evaluate whether the observed PFS benefit in patients with positive PD‐L1 expression translates into a significant improvement in OS. Additionally, the heterogeneity of NSCLC, with diverse subtypes and molecular alterations, may influence the efficacy of treatment. The development of resistance mechanisms over time can further limit the long‐term benefit of these drugs, even if they initially demonstrate significant PFS improvements. Moreover, the impact of subsequent lines of therapy, which may vary in effectiveness, can also affect OS. These factors collectively may contribute to the observed discrepancy between PFS and OS benefits in this study.

Bevacizumab exhibits immunomodulatory effects when combined with immune ICIs beyond its anti‐angiogenic properties.^29^ It promotes dendritic cell maturation, facilitating T cell activation, and enhances T cell infiltration into tumours by normalising tumour vasculature.^30^, ^31^ Moreover, it reduces myeloid‐derived suppressor cells (MDSCs) and Treg cell populations, fostering an immune‐permissive TME.^32^, ^33^ These effects synergise with ICIs to enhance their efficacy.

The optimal dosing regimen for bevacizumab in NSCLC remains an area of active investigation. While clinical guidelines offer varying recommendations, the AVAiL trial demonstrated improved PFS and OS with both 7.5 and 15 mg/kg bevacizumab in combination with chemotherapy compared with placebo.^34^ However, a post‐hoc analysis of Asian patients revealed a significant OS advantage with 7.5 mg/kg dose, associated with a lower incidence of severe adverse events.^35^ This suggests that a reduced dose of bevacizumab may offer a more favourable risk‐benefit profile for Asian NSCLC patients. Although direct comparisons between the two doses are limited by the absence of large‐scale randomised controlled trials, the phase III AVAiL trial provides additional support for the 7.5 mg/kg dose, demonstrating superior response rates. Furthermore, a retrospective real‐world study indicated no significant differences in efficacy or safety between the two dose groups.^36^ Considering these findings and the potential immunomodulatory effects of bevacizumab, the current study opted for a 7.5 mg/kg dose to achieve a balance between efficacy and safety, while also minimising treatment costs.^26^, ^28^

Osimertinib had been established as the standard first‐line modality for patients harbouring common *EGFR* mutations. However, in our study, patients previously treated with osimertinib had lower ORR and shorter PFS compared with those treated with first‐generation TKIs. Lam et al.^24^ reported that patients with acquired resistance to osimertinib experienced shorter PFS with the combination of atezolizumab, bevacizumab and chemotherapy compared with those without prior osimertinib exposure. Our previous retrospective study also identified prior osimertinib treatment as an adverse prognostic factor for patients treated with atezolizumab.^37^ This may be due to the fact that patients with prior osimertinib exposure often have a more advanced disease state, acquired resistance mutations such as T790M and/or having exhausted multiple lines of TKI therapy.

The phase II @Be study demonstrated the potential of combining bevacizumab and atezolizumab, achieving a 64% ORR in patients with PD‐L1 expression ≥50%, surpassing the 38.3% ORR with atezolizumab alone in high PD‐L1 expression subgroups of the IMpower110 study,^38^, ^39^ which further supported this synergy between ICIs and bevacizumab in IMpower 150 trial.^12^ In addition, HARMONi‐A trial also showed that ivonescimab, a PD‐1/VEGF bispecific antibody, in combination with chemotherapy significantly improved PFS compared with chemotherapy alone in patients with *EGFR*‐mutated NSCLC who had progressed on EGFR‐TKI treatment.^40^ These findings highlight the importance of bevacizumab's immunomodulatory effect, particularly in tumours with low immunogenicity, such as those with *EGFR* mutations.

The MARIPOSA‐2 study demonstrated a PFS benefit for amivantamab combined with chemotherapy and/or lazertinib in patients with *EGFR*‐mutated NSCLC who had progressed on osimertinib.^41^ While the amivantamab‐containing regimens exhibited superior ORRs and comparable PFS compared with the current study, distinct toxicity profiles were observed.^41^ Notably, infusion‐related reactions were more prevalent with amivantamab, and venous thromboembolism was a significant adverse event, particularly in patients receiving the triple combination of amivantamab, lazertinib and chemotherapy.^41^

ICIs offer survival benefits for various malignancies, but their efficacy is limited in *EGFR*‐mutated or *ALK* rearranged NSCLC cases.^11^, ^42^, ^43^ Robust biomarkers for predicting ICI response are lacking, prompting ongoing research. Studies explore diverse biomarkers including serum proteins, tumour‐specific receptors and TME factors.^44^, ^45^ Tumour‐infiltrating lymphocytes and PD‐L1 expression hold prognostic and predictive value in NSCLC patients receiving ICIs.^11^, ^46^ Our study confirms this, showing favourable response rates and longer PFS in tumours with PD‐L1 expression (≥1%). However, PD‐L1's predictive power remains limited due to assay variations and sample differences (fresh vs. archival).^47^, ^48^ Re‐biopsy ensured fresh tissue analysis, providing a more accurate reflection of PD‐L1 expression and the TME.

We found higher levels of peritumour NK cells, immune cells that fight cancer, in responders' tumours, suggesting their potential role in treatment success. NK cells, innate lymphoid cells with potent tumour‐killing and immune‐regulatory functions, have emerged as critical players in anti‐tumour immunity. The infiltration of NK cells into tumour tissue has been correlated with favourable prognostic outcomes.^49^, ^50^ Additionally, NK cells express various checkpoint molecules, including killer cell immunoglobulin‐like receptors, lymphocyte activation gene‐3 and PD‐1.^51^ Notably, activated NK cells, as a major source of IFN‐γ, contribute to tumour‐associated inflammation and upregulate PD‐L1 expression on tumour cells, promoting the regression of ‘hot’ tumours within an allergic inflammatory environment.^49^ PD‐1 expression on NK cells has been linked to diminished responsiveness. In mouse lymphoma models, PD‐1 impairs NK cell‐mediated immune surveillance, enabling tumour cell escape.^52^, ^53^ In metastatic melanoma patients, responders to anti‐PD‐1 ICIs exhibited significantly higher densities of intra‐ and peritumoural CD16^+^ and granzyme B^+^ NK cells.^54^ Therefore, inhibition of the PD‐1/PD‐L1 axis through anti‐PD‐1/PD‐L1 therapy can alleviate the suppressive effects of the TME on NK cell function, thereby enhancing their cytotoxic activity and promoting tumour cell killing. This also aligns with the development of new NK cell‐based therapies.

Exploring peripheral blood‐based biomarkers for ICI response is gaining traction due to their non‐invasive nature and potential for serial monitoring. However, clinically validated biomarkers are lacking. Our study found a significant link between pre‐treatment peripheral blood helper T cell levels and PFS. Patients with lower percentage of helper T cell before treatment had longer PFS. Helper T cells, though often considered secondary players in antitumour immune responses, play a crucial role in orchestrating effective immunity. Helper T cells not only enhance the B cell response but also facilitate the cytotoxic T lymphocyte (CTL) response.^55^ Promoting the activity of CTLs within a tumour by helper T cells plays a crucial role in cancer immunotherapy.^55^, ^56^ Without this support, memory CTLs exhibit dysfunctional phenotypes characterised by the expression of inhibitory receptors.^57^, ^58^ Recent studies have highlighted the pivotal role of helper T cells in initiating and sustaining anti‐PD‐1 immunotherapy responses.^56^ Furthermore, previous research has demonstrated that pretreatment CD4^+^ T cell counts, particularly those with a memory phenotype or high differentiation level, are associated with favourable treatment outcomes and prolonged PFS.^59^, ^60^ Inomata et al.^61^ reported a correlation between an early increase in PD‐1^+^CD4^+^ T cells after initiating ICIs and improved PFS in NSCLC patients. While our study did not differentiate based on CD27/CD28 expression, other research suggests specifics the CD4^+^ T cell subsets may predict treatment response and longer PFS in immunotherapy patients.^59^, ^62^ Given their critical role in immune responses, further research is needed to understand the role of systemic CD4^+^ T cell immunity in predicting treatment response.

Our study also showed a counterintuitive increase in circulating S100A9^+^ MDSCs following disease progression, aligning with prior studies.^63^, ^64^ These results suggest that profiling peripheral blood immune cell dynamics could predict treatment efficacy and patient prognosis in ICIs therapy. Targeting S100A9^+^ MDSCs may overcome acquired resistance to immunochemotherapy and anti‐angiogenesis agents.

Kang et al.^65^ reported that higher Tregs cells before ICIs correlated with poor ICI response. However, our study did not identify specific baseline immune cells predicting the combination treatment response. Discrepancies may stem from differences in treatment regimens, particularly the combined therapy employed here, enhancing ICIs effects. Additionally, antiangiogenic drugs reprogrammed immunosuppressive TMEs to supportive ones, potentially diminishing the predictive effect of Tregs.^60^, ^65^

The study's limitations include small sample size, single‐arm design and a homogeneous population. Larger phase III trials are needed for broader validation. Additionally, limited sample size for immune cell studies restricts immune profiling exploration. However, the study identified pre‐treatment peritumour NK cell infiltration, circulating S100A9^+^ MDSC and Tregs as potential biomarkers, suggesting the promise of immune cell profiling for personalised treatment with combined immunotherapy and chemotherapy. Further research is needed for validation.

In conclusion, this study demonstrates the promising efficacy of combining atezolizumab, bevacizumab, pemetrexed and cisplatin/carboplatin in *EGFR*‐mutated NSCLC patients who progressed on TKI therapy. Positive PD‐L1 expression (≥1%) correlated with higher ORRs and longer PFS. Furthermore, immune cell profiling may aid in identifying patients who may benefit from this approach.

## 4 MATERIAL AND METHODS

### 4.1 Study approved

The study adhered to the ethical principles of the Declaration of Helsinki and obtained approval from the Institutional Review Board of the National Taiwan University Hospital (REC Nos. 201908090MIFA). It was registered at ClinicalTrials.gov under the identifier NCT04147351. Prior to treatment initiation, all participating patients provided informed consent for molecular analyses. Interim analysis results were presented at ELCC 2022.^66^

### 4.2 Study design

This single‐centre, single‐arm phase II trial enrolled stage IIIB‐IV NSCLC patients with activating *EGFR* mutations after acquired resistance to EGFR TKIs. Lung cancer staging followed the eighth edition of the International Association for the Study of Lung Cancer TNM system.^67^

After screening for bone marrow function, organ function and measurable target lesions on CT scans, patients underwent *EGFR* genotyping on re‐biopsied samples. Patients with re‐biopsyed tissue samples without *EGFR^T790M^* mutations detected were enrolled. Additionally, PD‐L1 immunohistochemical (IHC) stain was performed using validated assays (Dako 22C3 or Ventana SP263).

The major exclusion criteria were history of previous exposure to platinum‐based chemotherapy, anti‐angiogenesis or ICIs (Supplementary Study Protocol). Patients with untreated symptomatic brain metastases or leptomeningeal disease were excluded. The patients whose re‐biopsy tissue showed *EGFR^T790M^* or exon20 insertion were also excluded because these tumour progression was still dependent on *EGFR* activation, which had less response to ICIs.

In the induction phase, patients received atezolizumab 1200 mg, bevacizumab 7.5 mg/kg, pemetrexed 500 mg/m^2^ and cisplatin 75 mg/m^2^ or carboplatin (creatinine clearance < 60 mL/min) AUC 5 mg/mL/min intravenously every 3 weeks for four cycles.

In the maintenance phase, patients continued atezolizumab, bevacizumab and pemetrexed. Bevacizumab and pemetrexed continued until disease progression, unacceptable toxicity or death. Atezolizumab could continue beyond radiographic progression by Response Evaluation Criteria in Solid Tumours (RECIST) version 1.1 if the investigator judged clinical benefit.

### 4.3 Disease monitoring and treatment efficacy evaluation

The primary endpoint of this investigation was ORR. Secondary endpoints comprised PFS, DoR and OS. Tumour imaging by chest, abdomen and brain CT was performed at baseline, every 6 weeks for the first 18 weeks, then every 9 weeks through the first 12 months, and every 12 weeks thereafter. Brain magnetic resonance imaging (MRI) was also an acceptable alternative brain imaging modality. Response assessments were conducted according to RECIST version 1.1, and treatment decisions were determined by the investigator.

PFS was defined as the time from date of enrolment to radiographically documented progression by RECIST version 1.1 or death from any cause (whichever occurred first). Participants alive and progression‐free, or lost to follow‐up, were censored at their last radiographic assessment. DoR was defined as the time from documented tumour response to radiographic disease progression or death. OS was measured from date of enrolment to death from any cause.

### 4.4 Safety outcome measures

During treatment, vital signs, physical examinations and urine protein dipstick tests were performed. Comprehensive serum chemistry panels complete blood counts with differentials were assessed every 3 weeks. Thyroid function tests were assessed at baseline, Week 3 and every 12 weeks thereafter. Patient survival were monitored every 8 weeks during the follow‐up phase.

Adverse events were reviewed. All adverse events and laboratory abnormalities were evaluated and graded according to the National Cancer Institute Common Terminology Criteria for Adverse Events (version 5.0).

### 4.5 Explore the TME by opal multiplex immunofluorescence staining

We employed an innovative multiplexed IHC imaging technique (Opal 6‐Plex Manual Detection Kit; Akoya Biosciences®). We performed tissue pre‐treatment and antibody incubation according to a previous publication,^68^, ^69^ but we replaced the secondary antibody with the EnVision plus detection system (Dako # K5007). The slides were washed, and the designated tyramide signal amplification dye (Opal 6‐Color kit; Akoya Biosciences®) was applied for 10 min according to the manufacturer's instructions. The slides were heated in retrieval buffer in a steamer to strip primary and secondary antibodies and pretreat the next staining target. Then, the cool down, blocking and antibody and Opal dye incubation steps were repeated for five more staining cycles. The primary antibodies used in the Lymphocytic Panel for separate incubation were as follows: CD4 (Abcam Cat# ab133616, RRID:AB_2750883, 1:1500); CD56 (Dako Cat# M7304, RRID:AB_2750583, 1:100); CD8 (Dako Cat# M7103, RRID:AB_2075537, 1:100); granzyme B (Monosan Cat# MON7029C, 1:500); FoxP3 (Biolegend Cat# 320102, RRID:AB_430881, 1:1000); and PanCK (Dako Cat# M3515, RRID:AB_2132885, 1:100). For the Immune Checkpoint Panel, staining will be followed by the following antibodies, including PD‐L1 (Cell Signaling Technology Cat# 13684, RRID:AB_2687655, 1:250), CD8 (Dako Cat# M7103, RRID:AB_2075537, 1:100), PD‐1 (Cell Signaling Technology Cat# 86163, RRID:AB_2728833, 1:200), CD163 (Leica Biosystems Cat# NCL‐L‐CD163, RRID:AB_2756375, 1:100), CD68 (Dako Cat# M0876, RRID:AB_2074844, 1:500) and PanCK (Dako Cat# M3515, RRID:AB_2132885, 1:100) (Tables S1 and S2). We calculated the immune cells in the tumour area, which was defined as an immune cell density.

### 4.6 Peripheral immune cell analysis

Additionally, the study investigated the peripheral immune cells and explored potential biomarkers for immunotherapy. Prior to treatment initiation, peripheral blood was collected from enrolled patients to identify potential immunotherapy biomarkers. Peripheral blood was collected before Cycle 1 (Day 0), post‐Cycle 1 (Week 2 ± 1), post‐Cycle 2 (Week 6), post‐Cycle 4 (Week 12) and after disease progression. Flow cytometry‐based comprehensive immune phenotyping of peripheral blood mononuclear cells (PBMCs) was performed. PBMCs were isolated using density‐gradient centrifugation and stained with various antibodies for analysis which were purchased from BioLegend (Table S3). Immune suppressor cells, including MDSCs and Treg cells, were also evaluated. The surface markers of immune cell populations were listed in Table S4. Furthermore, patients were stratified into low and high immune cell groups based on the median peripheral immune cell count to evaluate the association with PFS.

### 4.7 Statistics

Patient demographic characteristics and adverse event frequencies were summarised using descriptive statistical methods. All patients who received at least one dose of the study drug were eligible for safety evaluation. To ensure accurate reporting, patients were counted only once per preferred term, system organ class and individual patient.

According to the prior reports about the combination regimens for *EGFR* mutation‐positive patients, the range of the response rate was 43.9–70.6%.^12^, ^21^, ^24^, ^27^ We adopted a clinically relevant response rate of 50% to determine the necessary sample size, and Simon's optimal two‐stage design was used.^70^ Twenty participants were calculated for a type I error of 0.2 and power of 0.8, with an initial enrolment of six patients. If one or fewer responses were seen, the trial would end. Otherwise, 14 more patients would be added for a total of 20. If seven or fewer responses were observed by the second stage, the null hypothesis would be maintained, halting further investigation. Factoring in a 10% dropout rate, 22 patients were targeted for enrolment. Kaplan–Meier method was used to calculate PFS, OS and DoR with two‐sided 95% confidence intervals (95% CIs).

A non‐parametric Mann–Whitney *U* test was used to compare the proportions of peripheral immune cells between groups (CR/PR vs. SD) across different time points. Finally, serial measurements of peripheral blood immune cells were analysed using mixed‐effects ANOVA to assess the temporal effect (changes over time).

## AUTHOR CONTRIBUTIONS

S. G. W. and J. Y. S. designed the study and analysed the data. S. G. W. and J. Y. S. wrote and edited the manuscript. S. G. W., C. C. H., J. C. H. Y., B. C. L., C. Y. Y., Y. T. L., C. J. Y., W. Y. L. and J. Y. S. enrolled the patients and collect clinical data. S. H. Y., K. Y. Y. and W. S. K. performed Opal multiplex immunofluorescence staining and analysis immune cells in tumour samples. Y. F. L. and S. C. L. performed statistical analysis about the serial dynamic changes of peripheral immune cells. Y. T. C. was a consultant to contributed the flowcytometry analysis of peripheral immune cells. All the authors have approved the manuscript. The work reported in the paper has been performed by the authors, unless clearly specified in the text.

## CONFLICT OF INTEREST STATEMENT

S. G. W. received speaking honoraria from Takeda, Amgen, AstraZeneca, Pfizer, Roche, Chugai Pharma, Boehringer Ingelheim, Janssen, Novartis and Eli Lilly. C. C. H. has received grants from Astra Zeneca; and honorariums/speaker fees from Boehringer Ingelheim, Eli Lilly, Roche/Genentech/Chugai, MSD, Pfizer, Novartis, Bristol‐Myers Squibb and Ono Pharmaceutical. J. Y. S. has served as an advisory board member from Amgen, AstraZeneca, Roche, Pfizer, Novartis, Merck Sharp & Dohme, Takeda, CStone Pharmaceuticals, Janssen and Bristol‐Myers Squibb; received speaking honoraria from ACT Genomics; Amgen, Chugai Pharma, CStone Pharmaceuticals, Bayer, AstraZeneca, Eli Lilly, Boehringer Ingelheim, Genconn Biotech, Roche, Novartis, TTY Biopharm, Pfizer, Orient EuroPharma, MundiPharma, Takeda, Janssen, Merck Sharp & Dohme and Bristol‐Myers Squibb; and received a grant from F. Hoffmann‐La Roche Ltd. W. Y. L. has served as an advisory board member and received speaker's bureau from AstraZeneca, Roche, Boehringer Ingelheim, Eli Lilly, Pfizer, MSD Oncology, Novartis, Bristol‐Myers Squibb and Chugai Pharma Taiwan. J. C. H. Y. has received research grant from AstraZeneca; institutional fee for advisory or consultancy services from Amgen, AstraZeneca, Bayer, Boehringer Ingelheim, Bristol‐Myers Squibb, Daiichi Sankyo, Eli Lilly, Merck, Merck Sharp & Dohme, Novartis, Roche/Genentech, Takeda Oncology, Yuhan Parmaceuticals and JNJ; advisory or consultancy services from Ono Pharmaceuticals and Pfizer; and institutional fee for advisory services from advisory services from Puma Technology, Gilead and GSK. Y. T. L. has received speaking honoraria from ACT Genomics, AstraZeneca, Boehringer Ingelheim, Bristol‐Myers Squibb, Chugai Pharmaceutical, Eli Lilly, Illumina, Janssen, Manudipharma, Merck, Merck Sharp & Dohme, Novartis, Pfizer, Roche and Takeda; and support for attending meetings from Bristol‐Myers Squibb, Novartis and Takeda. C. Y. Y. has received speaking honoraria from AstraZeneca, Roche, Boehringer Ingelheim, Pfizer, Novartis, Bristol‐Myers Squibb, Ono Pharmaceutical, Merck Sharp & Dohme and Eli Lilly. All other authors declare no conflicts of interest.

## ETHICS STATEMENT

The study was conducted in accordance with the guidelines of the Declaration of Helsinki and approved by the Institutional Review Board of the National Taiwan University Hospital (REC Nos. 201908090MIFA and 202001101RIPB). All enrolled patients signed informed consent for molecular analyses before obtaining the tissue specimens.

## Supporting information



Supporting Information

## Data Availability

The datasets analysed during the current study are available from the corresponding author upon reasonable request.
