# Supplementary material for: Atezolizumab, bevacizumab, pemetrexed and platinum for EGFR‐mutant NSCLC patients after EGFR TKI failure: A phase II study with immune cell profile analysis
Source: Clin Transl Med. 2024 Dec 23;15(1):e70149. doi: 10.1002/ctm2.70149 (PMC11666332; doi:10.1002/ctm2.70149)
Supplement: Supplementary file 1 — Supporting Information [file CTM2-15-e70149-s001.pdf]

Supplementary Table S1 Characteristics of multiplex immunofluorescence panels.

Panel	Antibody	Clone	Vendor	Catalog number	RRID	Retrieval Buffer	Antibody dilution	Fluorophore	Fluorophore dilution
Lymphocytic Panel	CD4	EPR6855	Abcam	ab133616	AB_2750883	HTTR	1:1500	520	1:1000
	CD56	123C3	Dako	M7304	AB_2750583	EDTA	1:100	650	1:1000
	CD8	C8/144B	Dako	M7103	AB_2075537	HTTR	1:100	570	1:1000
	Granzyme B	GrB-7	Monosan	MON7029C		EDTA	1:500	620	1:500
	FoxP3	206D	Biolegend	320102	AB_430881	HTTR	1:1000	540	1:1000
	PanCK	AE1/AE3	Dako	M3515	AB_2132885	Citrate	1:100	690	1:1000
Immune Checkpoint Panel	PD-L1	E1L3N	CST	13684	AB_2687655	EDTA	1:250	520	1:1000
	CD8	C8/144B	Dako	M7103	AB_2075537	HTTR	1:100	570	1:1000
	PD-1	D4W2J	CST	86163	AB_2728833	EDTA	1:200	540	1:1000
	CD163	10D6	Leica	NCL-L-CD163	AB_2756375	HTTR	1:100	650	1:1000
	CD68	PG-M1	Dako	M0876	AB_2074844	EDTA	1:500	620	1:500
	PanCK	AE1/AE3	Dako	M3515	AB_2132885	Citrate	1:100	690	1:1000

Supplementary Table S2 The surface markers of the different immune cells detected by Opal Multiplex Immunofluorescence Staining

lymphocytic panel

Cell Subpopulations	CD4	CD56	CD8	Granzyme B	FoxP3
$CD4^+$ T_h	+	-	-	-/+	-/+
$CD4^+$ T_c	+	-	-	-/+	-/+
$CD4^+$ T_{reg}	+	-	-	-	+
Granzyme B^+ T_{reg}	+	-	-	+	+
$CD8^+$ T_c	-	-	+	-/+	-/+
Activated $CD8^+$ T_c	-	-	+	+	-
$CD8^+$ T_{reg}	-	-	+	-	+
Granzyme B^+ $CD8^+$ T_{reg}	-	-	+	+	+
$CD56^+$ NK	-	+	-/+	-/+	-
Activated $CD56^+$ NK	-	+	-	+	-
$CD8^+$/$CD56^+$ NKT	-	+	+	-/+	-
Activated $CD8^+$/$CD56^+$ NKT		+	+	+	-

Immune-checkpoint panel

Cell Subpopulations	CD8	CD68	CD163	PD-1	PD-L1
Exhausted $CD8^+$ Cells	+	-	-	+	-
Macrophage	-	+	-/+	-	-/+
M1 Macrophage	-	+	-	-	-/+
PD-$L1^+$ M1 Macrophage	-	+	-	-	+
M2 Macrophage	-	+	+	-	-/+
PD-$L1^+$ M2 Macrophage	-	+	+	-	+

Supplementary Table S3. Antibodies for Flow Cytometry

Brand	Antibody item	Vendor	Identifier (Catalog#)	RRID	Dilution
T、B、NK cell	CD3	BioLegend	300318	AB_314054	1:200
	CD4	BioLegend	300520	AB_389333	1:200
	CD8	BioLegend	301021	AB_2561281	1:200
	CD19	BioLegend	302216	AB_314246	1:200
	CD56	BioLegend	318306	AB_604101	1:200
	CD45	BioLegend	304036	AB_2561940	1:200
MDSC	CD14	BioLegend	301804	AB_314186	1:200
	CD11b	BioLegend	101262	AB_2572122	1:200
	S100A9	BioLegend	350705	AB_2564007	1:400
	HLA-DR	BioLegend	307616	AB_493588	1:200
	CD33	BioLegend	303416	AB_2561690	1:200
	PD-L1	BioLegend	329708	AB_940360	1:100
Memory T<br>Treg<br>PD-1<br>Tim3<br>Ki67	CD127	BioLegend	351328	AB_2562908	1:100
	CD25	BioLegend	302606	AB_314276	1:100
	CCR7	BioLegend	353236	AB_2563641	1:100
	CD39	BioLegend	328214	AB_2564575	1:100
	CD73	BioLegend	344024	AB_2650974	1:100
	PD-1	BioLegend	329950	AB_2566362	1:100
	Ki-67	BioLegend	350526	AB_2562872	1:100
	Tim-3	BioLegend	345016	AB_2561934	1:100
	CD45RA	BD	563870	AB_2738459	1:200
	FOXP3	Invitrogen	17-5773-82	AB_469457	1:200
pDC	Lineage	BioLegend	348801	AB_10612570	1:200
	CD123	BioLegend	306012	AB_439779	1:100
	CD304	BioLegend	354504	AB_11219194	1:100
	CD11c	BioLegend	301646	AB_2814122	1:100

Supplementary Table S4 The surface markers of the different immune cells

Phenotype	Leukocyte subsets
CD45+	total leukocytes
CD45+CD3+CD4+CD8-	Helper T cells
CD45+/CD3-CD19+	B cells
CD45+CD3+CD4-CD8+	Killer T cells
CD3+CD4+CD8-CCR7+CD45RA+	CD4 naïve
CD3+CD4+CD8-CCR7-CD45RA+	CD4 effector
CD3+CD4+CD8-CCR7+CD45RA-	CD4 central memory
CD3+CD4+CD8-CCR7-CD45RA-	CD4 effector memory
CD3+CD4-CD8+HLA-DR+	CD8 activated
CD3+CD4-CD8+CCR7+CD45RA+	CD8 naïve
CD3+CD4-CD8+CCR7-CD45RA+	CD8 effector
CD3+CD4-CD8+CCR7+CD45RA-	CD8 central memory
CD3+CD4-CD8+CCR7-CD45RA-	CD8 effector memory
CD3+CD4+CD8-CD25hiCD127lo FOXP3+	Treg
CD3+CD4+CD8-CD25hiCD127lo Foxp3+ CD39+	CD39+ Treg
CD3+CD4+CD8-CD25hiCD127lo Foxp3+ CD73+	CD73+ Treg
CD45+HLA-DR+Lin-CD123+CD304+CD11c-	plasmacytoid dendritic cells
CD45+CD3- CD56+	NK cells
CD45+/CD11b+/CD14+/S100A9+	MDSC

Supplementary Table S5 Treatment-related adverse events.

	Any grade	(%)	Grade >=3
**Abnormal liver function**	7	31.8%	2
**Neutropenia**	4	18.2%	1
**Constipation**	4	18.2%	0
**Rash acneiform**	4	18.2%	0
**Thrombocytopenia**	3	13.6%	1(ITP)
**UTI**	3	13.6%	1(Renal abscess)
**Pulmonary embolism/DVT**	2	9.1%	2
**Anemia**	2	9.1%	1
**Hypertension**	2	9.1%	0
**Dizziness**	2	9.1%	0
**Fever**	2	9.1%	0
**Insomnia**	2	9.1%	0
**URI**	2	9.1%	0
**Headache**	2	9.1%	0
**Nausea/Vomiting**	2	9.1%	0
**Hydrocephalus**	1	4.5%	1
**Gingivitis**	1	4.5%	0
**Malaise**	1	4.5%	0
**Dyspnea**	1	4.5%	0
**Muscle acne**	1	4.5%	0
**Leg edema**	1	4.5%	0
**Cellulitis**	1	4.5%	0
**Gout**	1	4.5%	0
**Diarrhea**	1	4.5%	0
**Anxiety**	1	4.5%	0
**Fatigue**	1	4.5%	0
**Epistaxis**	1	4.5%	0
**adrenal insufficiency**	1	4.5%	0
**Cough**	1	4.5%	0
**Back pain**	1	4.5%	0
**Eustachian tube obstruction**	1	4.5%	0
**Hyponatremia**	1	4.5%	0
**Oral mucositis**	1	4.5%	0
**Hemorrhoid**	1	4.5%	0
**Acute kidney injury**	1	4.5%	0
**Sore throat**	1	4.5%	0
**Hiccup**	1	4.5%	0
**Lower limb pain**	1	4.5%	0
**Low Back pain**	1	4.5%	0
**Rib pain**	1	4.5%	0

Supplementary Table S6 Treatment after disease progression.

**Progression on study treatment**	21		**(100%)**	
**Tissue Re-biopsy done**	13		(61.9%)	
Adenocarcinoma		12		(92.3%)
Small cell transformation		1		(7.7%)
**NGS-based comprehensive genomic profiling**	9		(42.9%)	
**Tissue-based versus Liquid**	6 versus 3			
EGFR T790M		0		(0.0%)
EGFR exon19 deletion		6		(66.7%)
EGFR L858R		2		(22.2%)
TP53 mutation		2		(22.2%)
BRCA2 mutation		2		(22.2%)
EGFR amplification		1		(11.1%)
Kras amplification		1		(11.1%)
DNMT3A mutation(W314*)		1		(11.1%)
PTEN loss		1		(11.1%)
CCDC6-RET fusion		1		(11.1%)
MET amplification		1		(11.1%)
ERBB2 amplification		1		(11.1%)
MSH6 mutation(splice acceptor		1		(11.1%)
CDKN2A homozygous deletion		1		(11.1%)
CCND1 amplification		1		(11.1%)
MDM2 amplification		1		(11.1%)
MYC amplification		1		(11.1%)
RICTOR amplification		1		(11.1%)
FGF19 amplification		1		(11.1%)
FGF3 amplification		1		(11.1%)
FGF4 amplification		1		(11.1%)
RAD21 amplification		1		(11.1%)
CDK4 amplification		1		(11.1%)
**Any systemic treatment**	18		(85.7%)	
**Lines of systemic treatment (median, range)**	2 (0-9)			
Chemotherapy	13		(61.9%)	
Rechallenge EGFR TKI	17		(81.0%)	
erlotinib		10		(58.8%)
osimertinib		7		(41.2%)
Antiangiogenesis	3		(14.3%)	
EGFR TKI+ antiangiogenesis		3		(100%)
**Radiotherapy**	10		(47.6%)	

Supplementary Figure Legend

Supplementary Figure S1 The heatmap of the comprehensive genomic profiling after disease progression.

Supplementary Figure S2 The distribution of peripheral immune cells at Day 0 according to treatment responses (CR/PR vs. SD) (A) T cell; (B) B cell; (C)NK cell; (D)Helper T cell; (E)Cytotoxic T cell; (F)S100A9 MDSC; (G) Plasmacytoid dendritic cells (pDCs) cell; (H) Regulatory T (Treg) cell; (I) CD39$^+$ Treg; (J) CD73$^+$ Treg; (K) CD39$^+$/CD73$^+$Treg; (L) CD4$^+$PD-1$^+$ cell; (M) CD4$^+$TIM3$^+$ cell; (N) CD4$^+$Ki67$^+$ cell; (O) CD8$^+$PD-1$^+$ cell; (P) CD8$^+$TIM3$^+$ cell; (Q) CD8$^+$Ki67$^+$ cell; (R) CD4$^+$ naïve T cell; (S) CD4$^+$ central memory T cell; (T) CD4$^+$ effector memory T cell; (U) CD4$^+$ effector T cell; (V) CD4$^+$ central memory cells/effector cells; (W) CD8$^+$ naïve T cell; (X) CD8$^+$ central memory T cell; (Y) CD8$^+$ effector T cell; (Z1) CD8$^+$ effector T cell; (Z2) CD4$^+$ central memory cells/effector cells. (CR/PR: Complete response/Partial response; SD: stable disease)

Supplementary Figure S3 The serial dynamic changes of peripheral immune cells (A) T cell; (B) B cell; (C)NK cell; (D)Helper T cell; (E)Cytotoxic T cell; (F)S100A9 MDSC; (G) Plasmacytoid dendritic cells (pDCs) cell; (H) Regulatory T (Treg) cell; (I) CD39$^+$ Treg; (J) CD73$^+$ Treg; (K) CD39$^+$/CD73$^+$Treg; (L) CD4$^+$PD-1$^+$ cell; (M) CD4$^+$TIM3$^+$ cell; (N) CD4$^+$Ki67$^+$ cell; (O) CD8$^+$PD-1$^+$ cell; (P) CD8$^+$TIM3$^+$ cell; (Q) CD8$^+$Ki67$^+$ cell; (R) CD4$^+$ naïve T cell; (S) CD4$^+$ central memory T cell; (T) CD4$^+$ effector memory T cell; (U) CD4$^+$ effector T cell; (V) CD8$^+$ naïve T cell; (W) CD8$^+$ central memory T cell; (X) CD8$^+$ effector T cell; (Y) CD8$^+$ effector T cell. Post-C1: Post-Cycle 1; Post-C2: Post-Cycle 2; Post-C4: Post-Cycle 4; PD: Disease progression.

Supplementary Figure S4 Pearson correlation analysis was used to assess the correlation between peripheral blood helper T cell counts and tumor tissue infiltration density in 12 patients with paired pre-treatment re-biopsy tissue and peripheral blood samples.

Supplementary Figure S1 The heatmap of the comprehensive genomic profiling after disease progression.

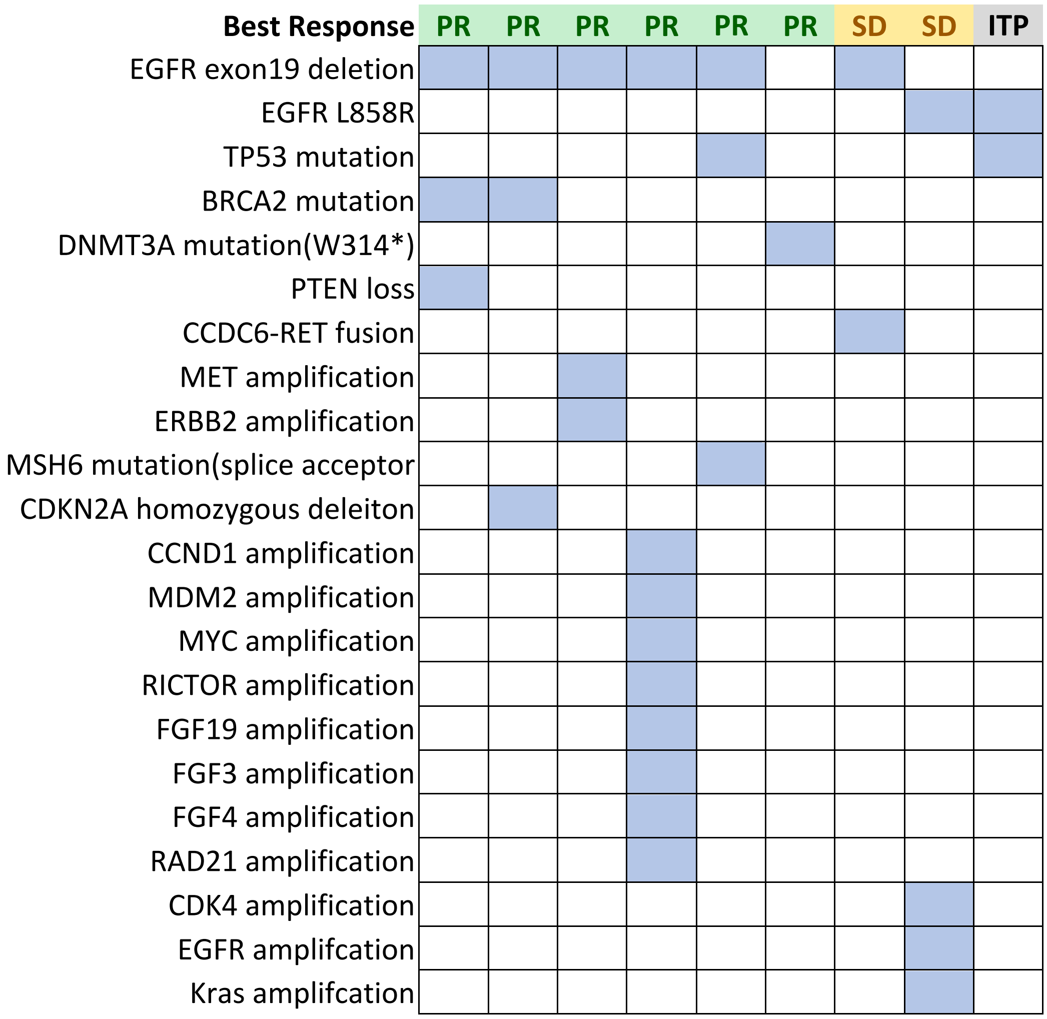

Supplemental Figure S2

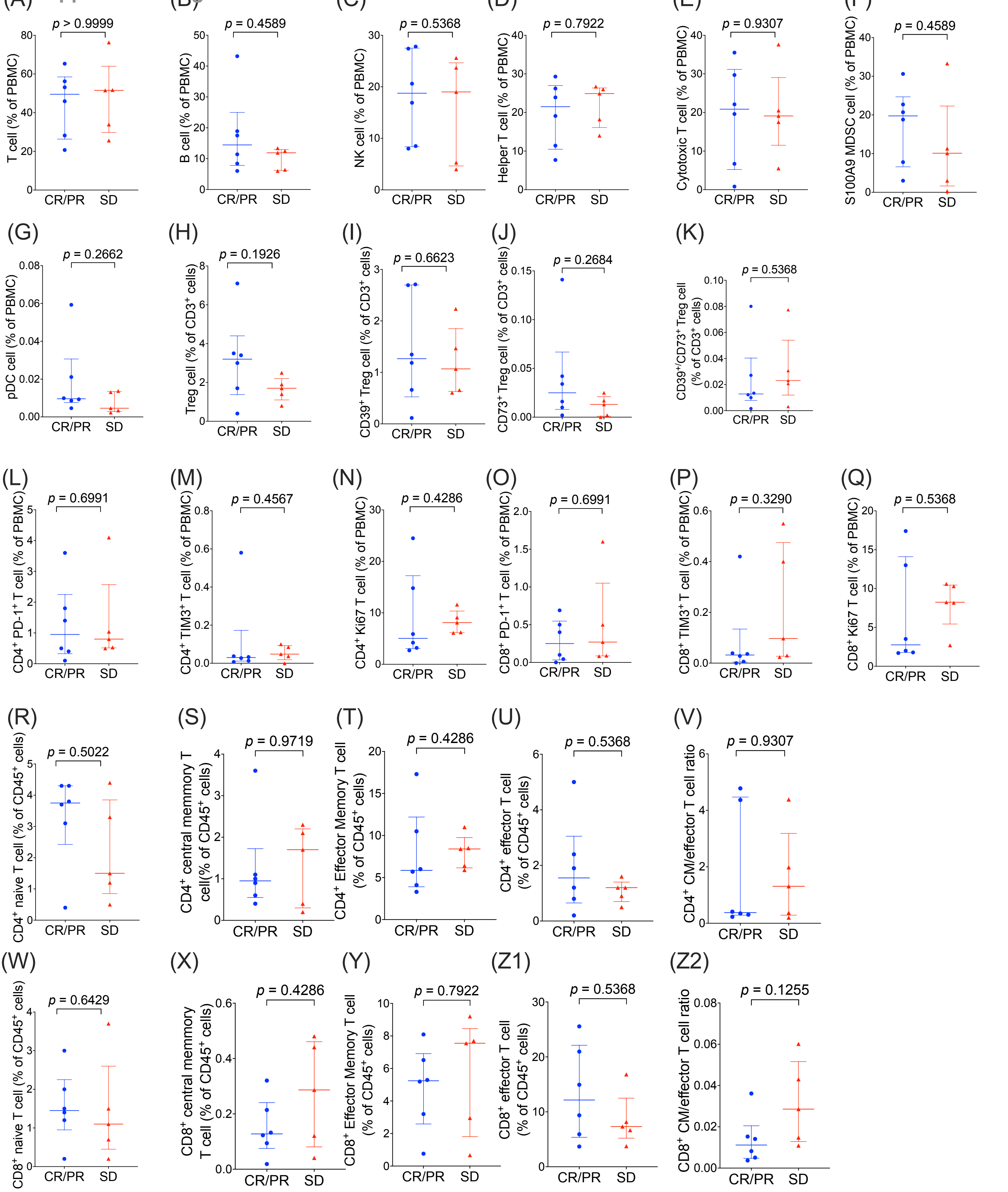

Supplemental Figure S3

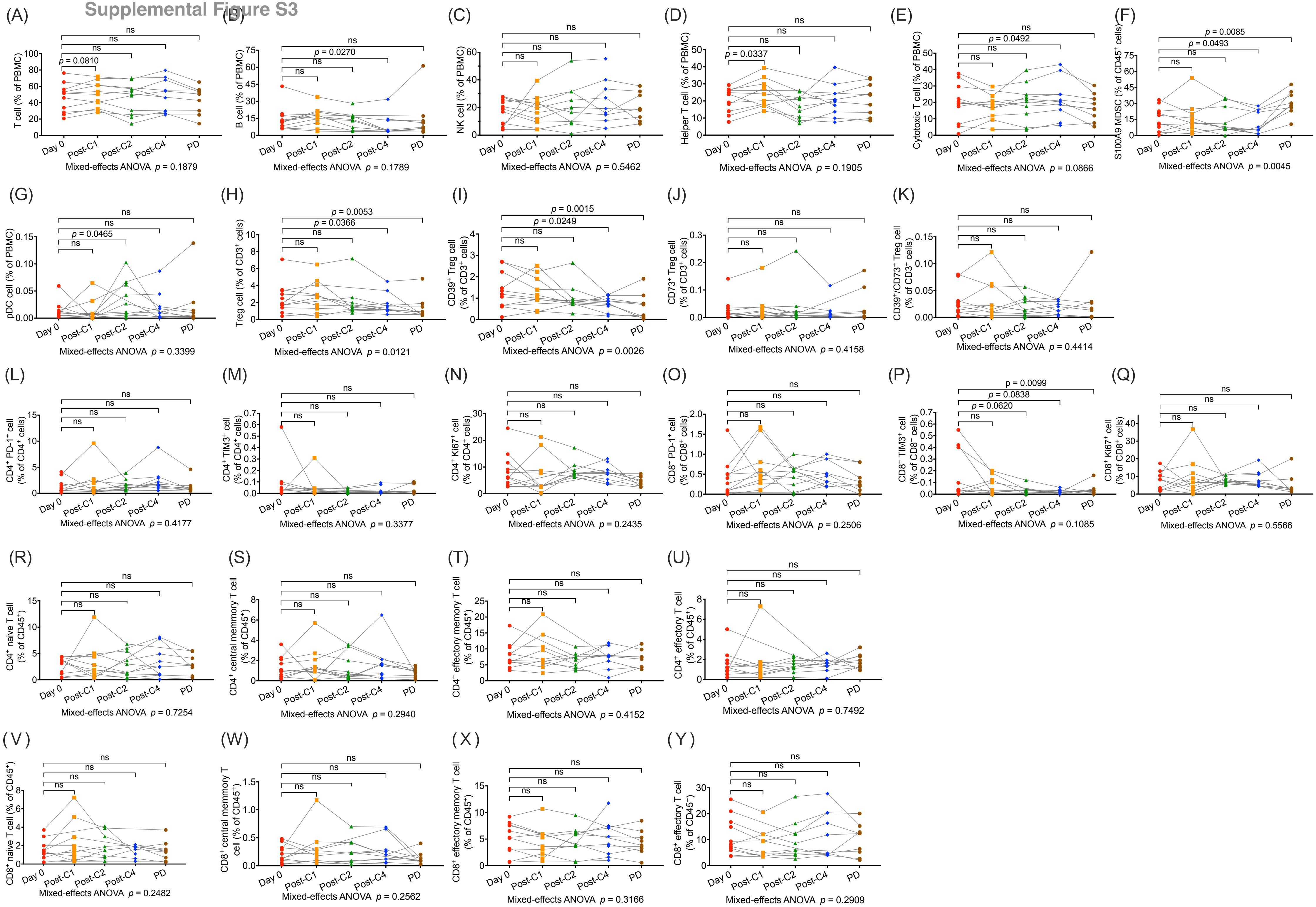

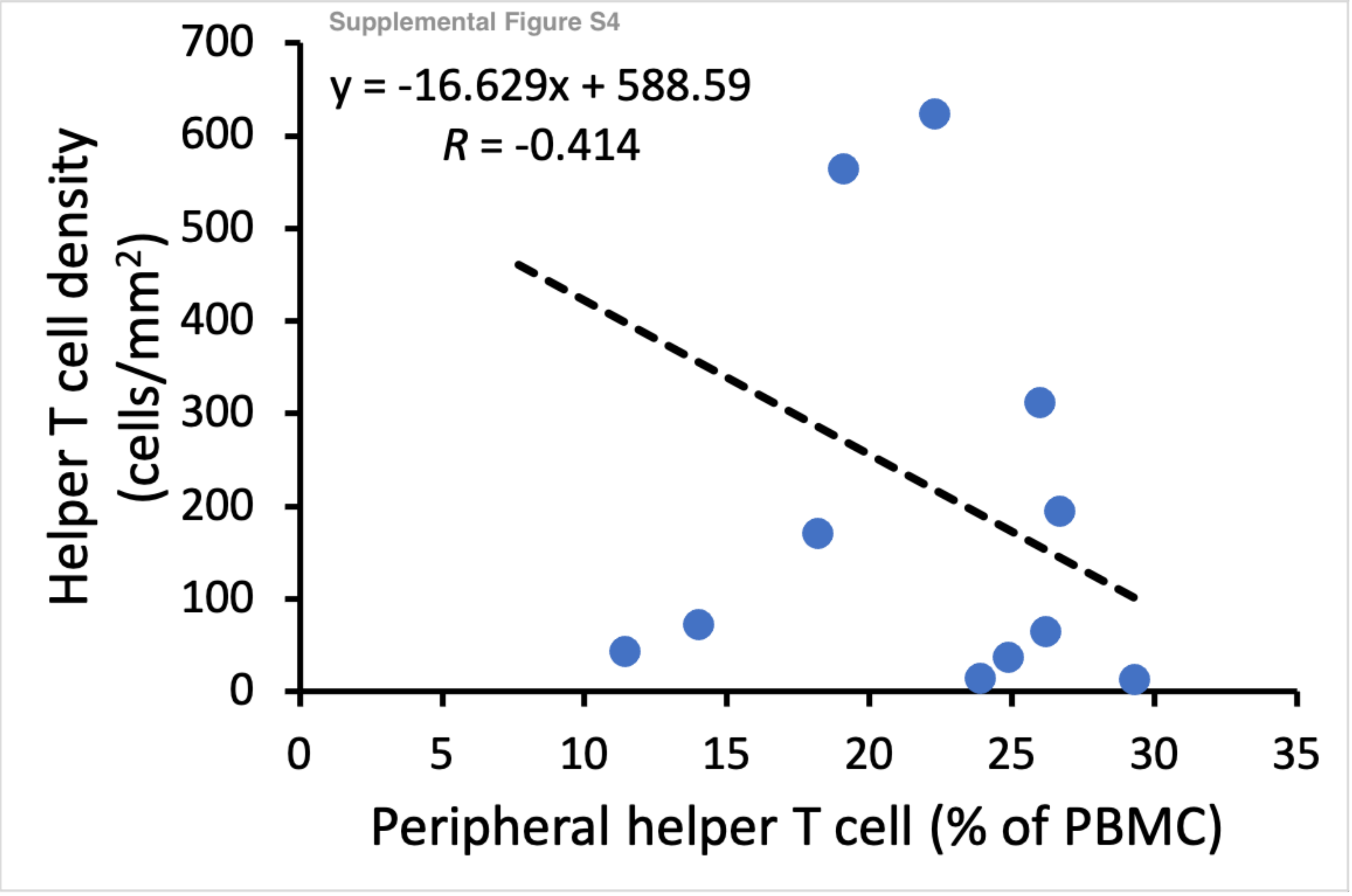

Supplemental Figure S4
y = -16.629x + 588.59
R = -0.414
Helper T cell density (cells/mm2)
700
600
500
400
300
200
100
0
0
5
10
15
20
25
30
35
Peripheral helper T cell (% of PBMC)
